# microRNA-569 inhibits tumor metastasis in pancreatic cancer by directly targeting NUSAP1

**DOI:** 10.18632/aging.204035

**Published:** 2022-04-28

**Authors:** Xiaohui Guo, Yatian Li, Xiaofang Che, Kezuo Hou, Xiujuan Qu, Ce Li

**Affiliations:** 1 Department of Medical Oncology, The First Hospital of China Medical University, Shenyang 110001, China; 2 Key Laboratory of Anticancer Drugs and Biotherapy of Liaoning Province, The First Hospital of China Medical University, Shenyang 110001, China; 3 Liaoning Province Clinical Research Center for Cancer, The First Hospital of China Medical University, Shenyang 110001, China; 4 Key Laboratory of Precision Diagnosis and Treatment of Gastrointestinal Tumors, Ministry of Education, The First Hospital of China Medical University, Shenyang 110001, China

**Keywords:** miR-569, NUSAP1, pancreatic cancer, metastasis, ZEB1

## Abstract

MicroRNAs (miRNAs) are known to be involved in the development and progression of pancreatic cancer (PC). In this study, the prognostic significance and mechanistic role of microRNA-569 in PC were explored. Quantitative real-time PCR was used to detect the expression of microRNA-569 in PC tissues and cell lines. Scratch test and Transwell assay were conducted to detect migration and invasion ability. The xenograft nude mice model was used to determine tumor metastasis *in vivo*. The direct targets of microRNA-569 were determined by using bioinformatics analysis and a dual-luciferase reporter assay. The expression level of microRNA-569 was down-regulated in PC patients with a poor prognosis. *In vitro* and *in vivo* experiments indicated that over-expression of microRNA-569 inhibited the migration and invasion of PC cells. MicroRNA-569 negatively regulated NUSAP1 by directly binding its 3'-untranslated region. Further mechanism research implied that the ZEB1 pathway was involved in microRNA-569/NUSAP1 mediation of the biological behaviors in PC. These data demonstrated that microRNA-569 may exert a tumor-suppressing effect in PC and maybe a potential therapeutic target for PC patients.

## INTRODUCTION

Pancreatic cancer is often referred as the “king of cancers”, with a very poor prognosis, and is the fourth leading cause of cancer death in the world [1]. The main causes for the poor prognosis are its high aggressiveness, early metastasis, and profound chemoresistance [2, 3]. Due to the lack of specific diagnostic methods, more than 80% of patients with PC were found to have locally unresectable or metastatic diseases [4]. Even for patients who underwent a successful operation or good response to chemotherapy, most patients will eventually have local recurrence or metastasis [5]. PC is prone to metastasize to the liver, lung, lymph nodes, and bones, which is closely related to patient death [6]. Thus, exploring the potential core gene affecting the malignant progression of pancreatic cancer is very crucial.

MicroRNAs (miRNAs), containing 22 to 25 nucleotides, belong to single-stranded ribonucleic acid and exert function by degrading or inhibiting the translation of other proteins to regulate gene expression [7]. Dysregulation of miRNAs were closely associated with the occurrence of various diseases, especially cancers [8–12]. In lung cancer, miRNA-569 can be used as a tumor suppressor to induce apoptosis [13]. In mammary cancer, over-expression of miRNA-569 has a poor prognosis, and it could inhibit cancer progression by down-regulating TP53INP1 [14], however, there are no reports available on the effect of miR-569 in PC.

Nucleolar and spindle-associated protein 1 (NUSAP1), mainly modulates spindle assembly and stability during mitosis [15]. Previous studies have reported abnormal NUSAP1 expression presenting in multiple malignant tumors. The expression of NUSAP1 was up-regulated in colon cancer, which demonstrated a poor prognosis [16]. Fang et al. that NUSAP1 was significantly up-regulated in renal cell carcinoma, which strengthened a series of malignant biological behaviors of tumor cells [17]. However, NUSAP1 is rarely reported in pancreatic cancer.

Our research is the first to investigate the miRNA-569 expression in PC and reveal its relation with clinical outcomes. Furthermore, the biological role of miRNA-569 and its potential molecular mechanisms were investigated. miRNA-569 directly targets NUSAP1, which in turn regulates ZEB1 expression and inhibits PC cell migration and invasion.

## RESULTS

### microRNA-569 is an indicator of good prognosis

**Figure 1:**
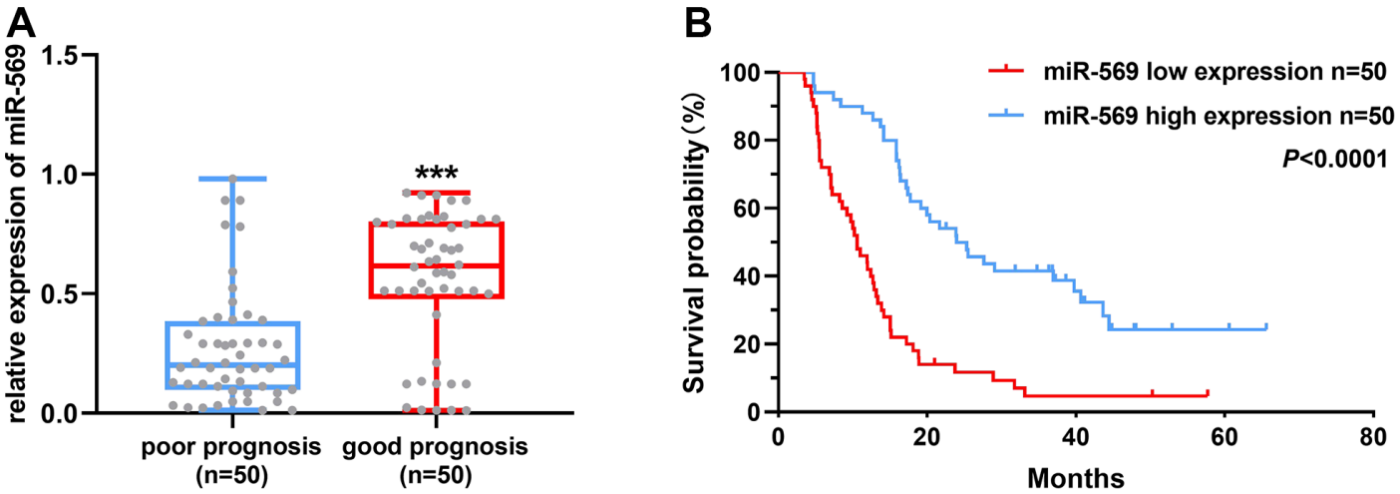
**miR-569 was down-regulated in PC tissues and patients with high miR-569 expression had a good prognosis.** (**A**) Analysis of miR-569 expression in PC tissues; (**B**) Kaplan-Meier Plotter analysis of the effect of miR-569 on PC patient survival.

Patients with pancreatic cancer were divided into the following two groups: poor prognosis, 12 months or less; and good prognosis, above 21 months according to the survival time. As shown in Figure 1A, the high miRNA-569 expression has a good prognosis, suggesting that miR-569 could serve as a good prognostic marker. Furthermore, the overall survival of PC patients with low miRNA-569 expression is shorter (Figure 1B). The median survival time of the low expression group was 10.6 months, while the high-expression group was 24.0 months.

### microRNA-569 over-expression suppresses migration and invasion in PC cells

**Figure 2:**
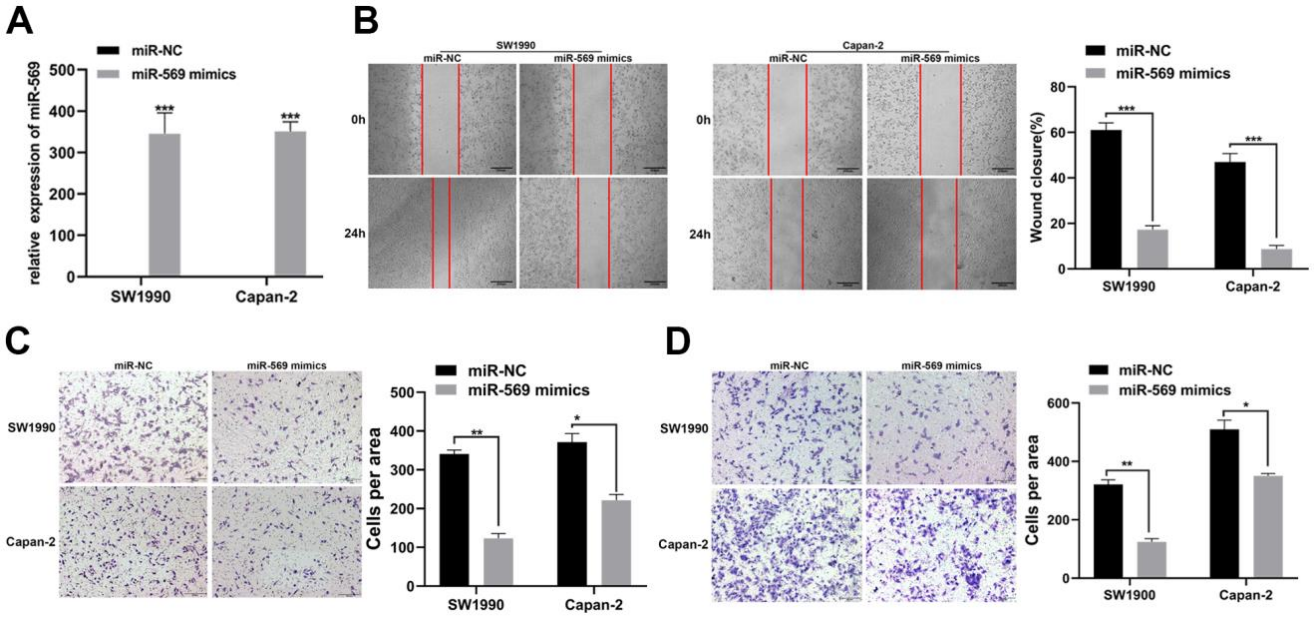
**miR-569 inhibited the migration and invasion of PC cells *in vitro*.** (**A**) RT-qPCR showed the miR-569 transfection efficiency. (**B**) Wound healing assay demonstrated migratory abilities of PC cells after over-expression of miR-569; (**C**) Transwell assay showed migratory abilities of PC cells after over-expression of miR-569; (**D**) Transwell assay indicated invasive abilities of PC cells after over-expression of miR-569. (* *p <* 0.05, ** *p <* 0.01, *** *p <* 0.001, *n* = 3, Student’s *t*-test, means ± 95% CI).

PC is prone to metastasis in the late stage, especially liver metastasis, leading to a poor prognosis. The expression levels of miR-569 after transfection of mimics in SW1990 and Capan-2 cells were confirmed. As expected, miR-569 levels were significantly up-regulated after transfection of mimics (Figure 2A). Scratch assay and Transwell experiment indicated that, compared to miR-NC, the exogenous increase of miRNA-569 expression can inhibit the migration of PC cells (Figure 2B, 2C). Furthermore, the Transwell invasion assay also helped to confirm that overexpression of miRNA-569 could inhibit cell invasion (Figure 2D), thus, these data revealed that miRNA-569 has an obvious anti-metastatic effect.

### microRNA-569 inhibits PC liver metastasis *in vivo*

**Figure 3:**
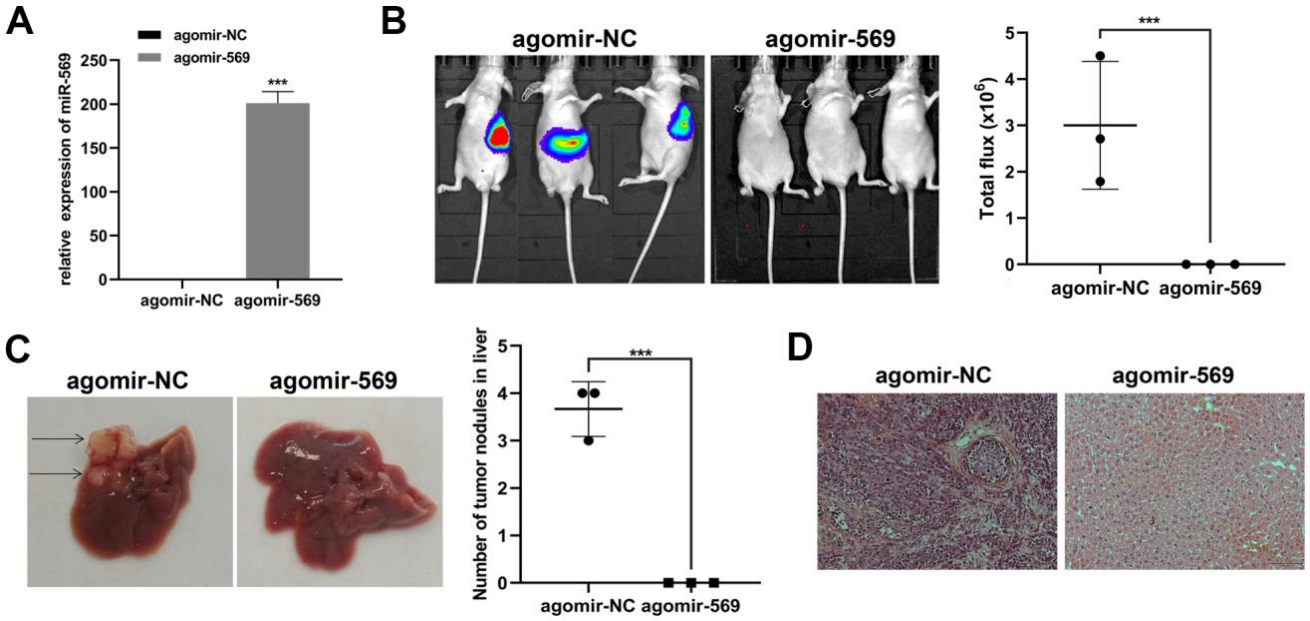
**miR-569 inhibited PC cell metastasis *in vivo*.** (**A**) RT-qPCR showed miRNA agomir transfection efficiency. (**B**) *In vivo* bioluminescence imaging of representative animals from each treatment group 10 weeks after tumor cell inoculation. (**C**) Representative images of the liver of nude mice. Black arrows show metastatic tumor colonies in the liver. (**D**) HE staining of metastatic tumor colonies in the liver, magnification ×100. (* *p <* 0.05, ** *p <* 0.01, n = 5, Student’s *t*-test, means ± 95% CI).

Then, we focus on the antitumor effect of miRNA-569 *in vivo*. Capan-2 was transfected with agomir-569 and control, respectively. Transfected cells were injected into the spleen of mice to establish the liver metastasis model, and the metastatic focus was evaluated ten weeks later (Figure 3A). *Ex vivo* luciferase imaging demonstrated that miRNA-569 overexpression significantly reduced liver metastasis in Capan-2 cells compared with negative control (Figure 3B). The number of metastatic foci in mice injected with agomir-569 cells decreased significantly (Figure 3C). As indicated in Figure 3D, the hepatic metastatic foci were stained by hematoxylin and eosin (HE). In summary, it can be inferred that miRNA-569 can inhibit liver metastasis of PC, which might have certain clinical significance.

### Prediction and screening of target genes

**Figure 4:**
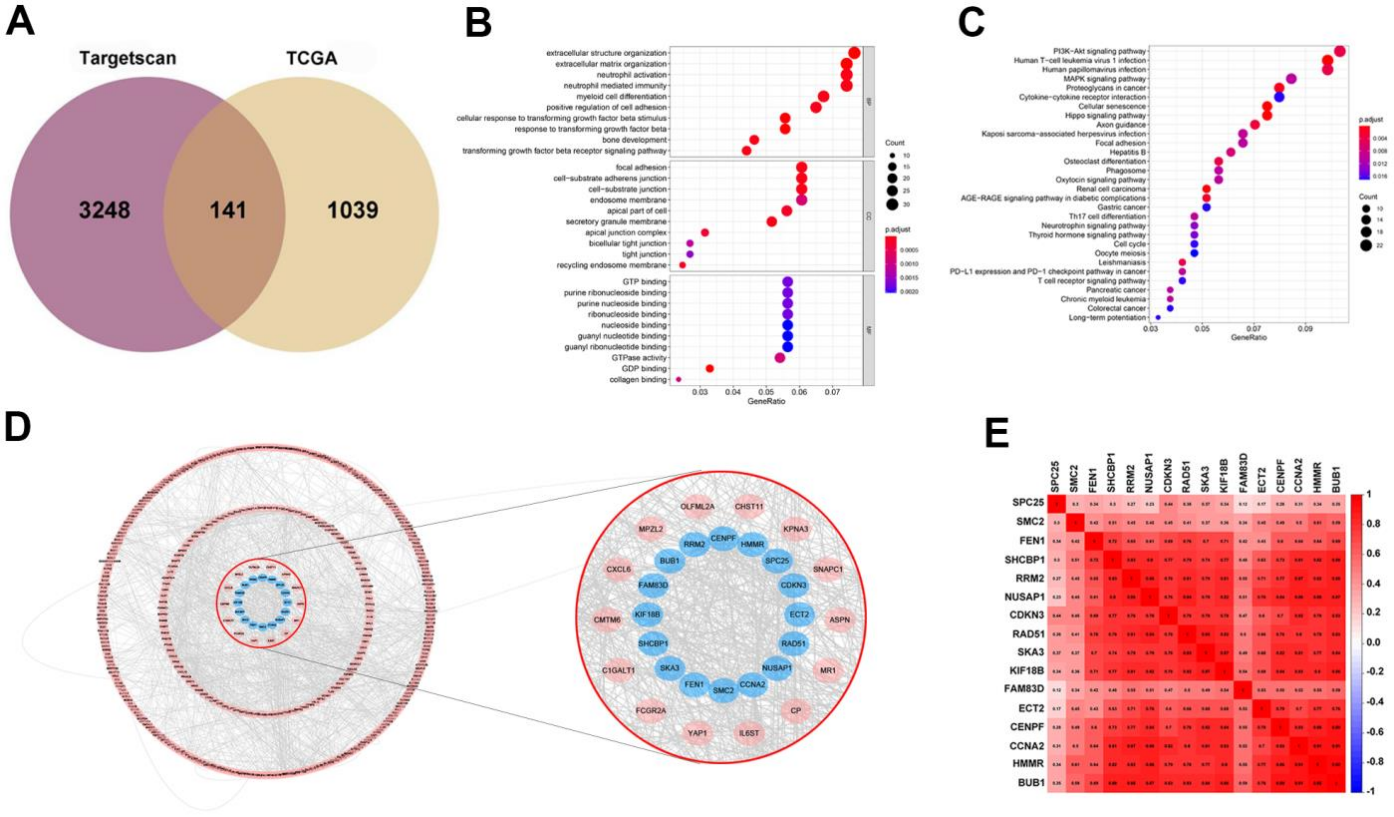
**Prediction and screening of the target gene of miR-569 through bioinformatics analysis.** (**A**) Venn diagram for the intersections between DEGs and miRNA target genes. (**B**, **C**) GO and KEGG analysis shows pathways in which the miR-569 target gene participates. (**D**) Protein-protein interaction networks of the miR-569 target genes using the Cytoscape database. (**E**) Correlation heat map of hub genes.

**Figure 5:**
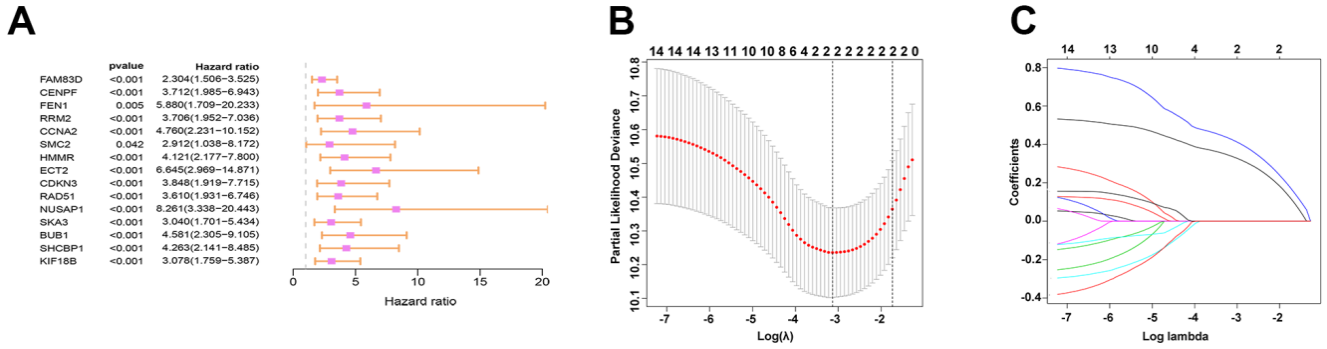
**Prediction and screening of the target gene of miR-569 through bioinformatics analysis.** (**A**) Forest plot for hazard ratios of survival-associated hub genes in PC. (**B**) Partial likelihood deviance versus log (Ḽ) was drawn using a LASSO Cox regression model. (**C**) Coefficients of selected features are shown in the terms of λ.

TargetScan was used for the prediction of the target genes. Then by interaction with up-regulated genes as evidenced by the TCGA database, 141 target genes were identified and viewed in the form of a Venn diagram (Figure 4A). GO and KEGG analyses were applied to clarify the function of miRNA-569. GO analysis is composed of Biological processes (BPs), cellular components (CCs), and molecular functions (MFs) [18], which mainly concentrated on the extracellular matrix organization, focal adhesion, and GTPase activity, which were critical in the processes of cell biology (Figure 4B). For KEGG analysis, the PI3K-Akt signaling pathway and MAPK signaling pathway were reported to be closely related to the cancerous process of PC (Figure 4C). To screen hub genes, we constructed a protein-protein interaction network that consists of 399 nodes and 1499 edges, and we then focused on the 16 hub genes in the network highlighted by cytoHubba according to their degree of overexpression (Figure 4D, 4E). Since miRNA-569 was closely associated with prognosis, the LASSO Cox analysis was used to limit the possible choices of hub genes to facilitate the selection of only the most useful target genes. This allowed us to identify two genes (ECT2 and NUSAP1) (Figure 5A–5C). The biological function of ECT2 in pancreatic cancer had been discussed in our previous work [19], so we mainly focused on NUSAP1 during subsequent research.

### Verification of target genes

**Figure 6:**
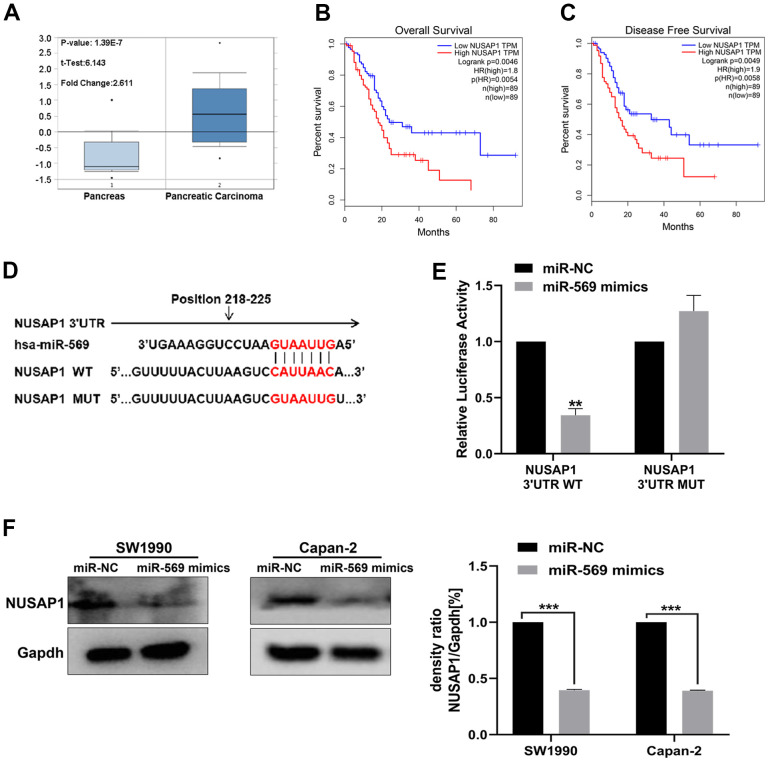
**miR-569 directly targeted NUSAP1.** (**A**) Oncomine database showing NUSAP1 mRNA expression level in PC and normal tissues. (**B**, **C**) Kaplan-Meier overall survival and disease-free survival curves for patients with PC stratified by high and low expression of NUSAP1. (**D**) Putative miR-569 target sequence in wild-type (WT) and mutated (MUT) 3’UTR of NUSAP1 was generated as indicated. (**E**) Relative luciferase activity of NUSAP1 3’UTR co-transfected with the indicated reporters and miR-569 mimic oligonucleotides. (**F**) Western blot assay demonstrated NUSAP1 protein level after over-pression of miR-569. (* *p <* 0.05, ** *p <* 0.01, *n* = 5, Student’s *t*-test, means ± 95% CI).

By analyzing data available within the Oncomine database, NUSAP1 was found to be up-regulated in PC tissues relative to normal control (Figure 6A). High expression of NUSAP1 significantly shortened the overall survival time and disease-free survival time (Figure 6B). The base pairing was observed between mature miR-569 and the 3’-UTR region of the NUSAP1 gene-seed sequence (Figure 6C). A dual-luciferase experiment was then performed: as shown in Figure 6D, for the NUSAP1-3’-UTR-WT group, compared with miR-NC, the luciferase activity of miR-569 mimics was weakened. However, there was no significant difference in the NUSAP1-3’-UTR-MT group. In addition, miR-569 could block the expression of NUSAP1 protein (Figure 6E).

### NUSAP1 knockdown suppresses PC cell migration and invasion

**Figure 7:**
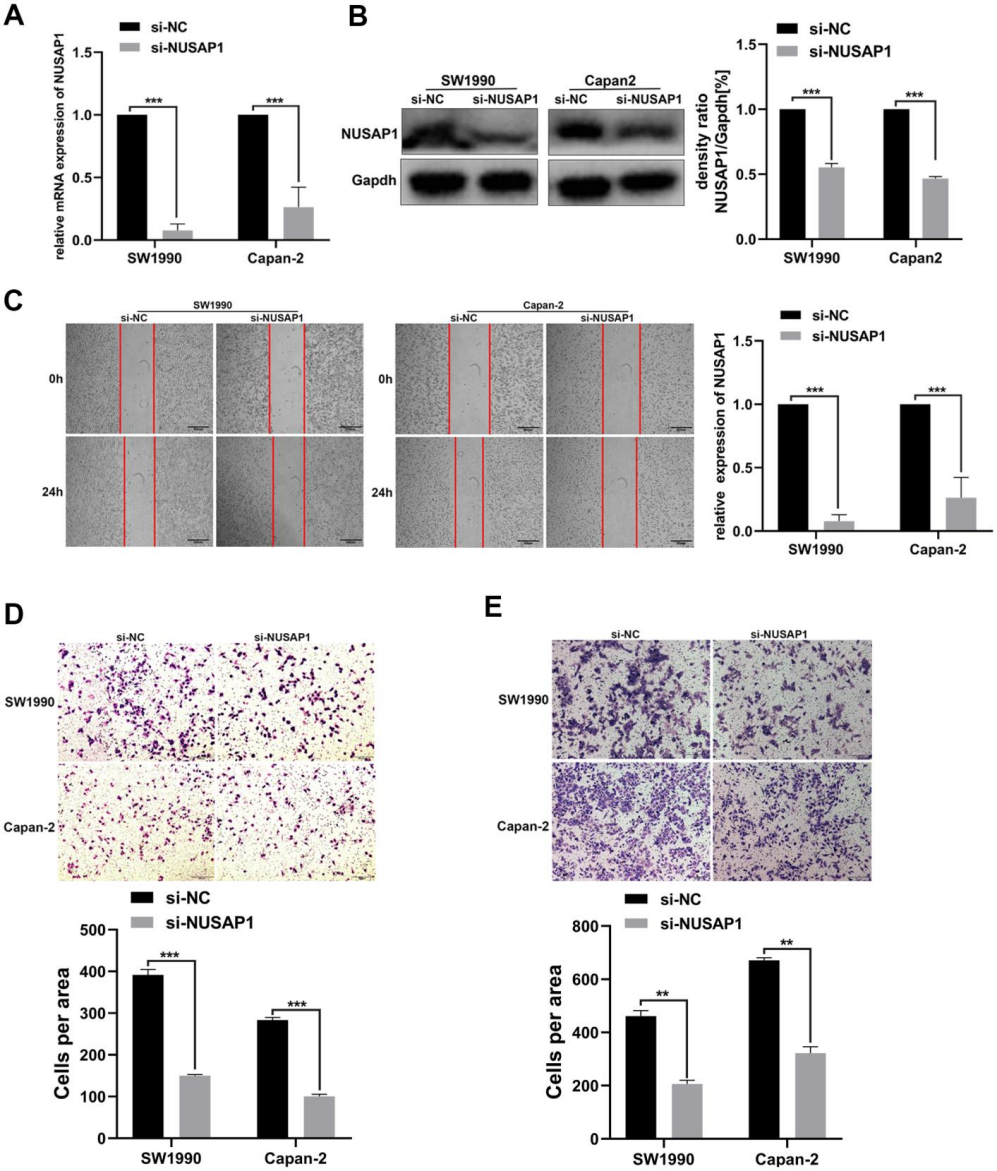
**NUSAP1 promoted the migration and invasion of PC cells.** (**A**) Western blot and (**B**) qRT-PCR were performed to verify transfection efficiency (*p < 0.05). (**C**) Wound healing assay showed migratory abilities of PC cells after knocking down NUSAP1; (**D**) Transwell assay indicated migratory abilities of PC cells after knocking down NUSAP1; (**E**) Transwell assay showed invasive abilities of PC cells after knocking down NUSAP1. GAPDH was used as a loading control in Western blot assay. (* *p <* 0.05, ** *p <* 0.01, *** *p <* 0.001, n = 3, Student’s *t*-test, means ± 95% CI).

Then, we explored the influence of changes in the expression of NUSAP1 on the malignant biological behavior of PC. PCR and Western blot assay were carried out to validate the knockdown efficiency (Figure 7A, 7B). As expected, NUSAP1 knockdown significantly inhibited PC cell migration (Figure 7C). In addition, Transwell experiment demonstrated that knockdown of endogenous NUSAP1 expression inhibited migration and invasion of SW1990 and Capan-2 (Figure 7D, 7E).

### The miRNA-569/NUSAP1/ZEB1 axis is involved in the metastasis of PC cells

**Figure 8:**
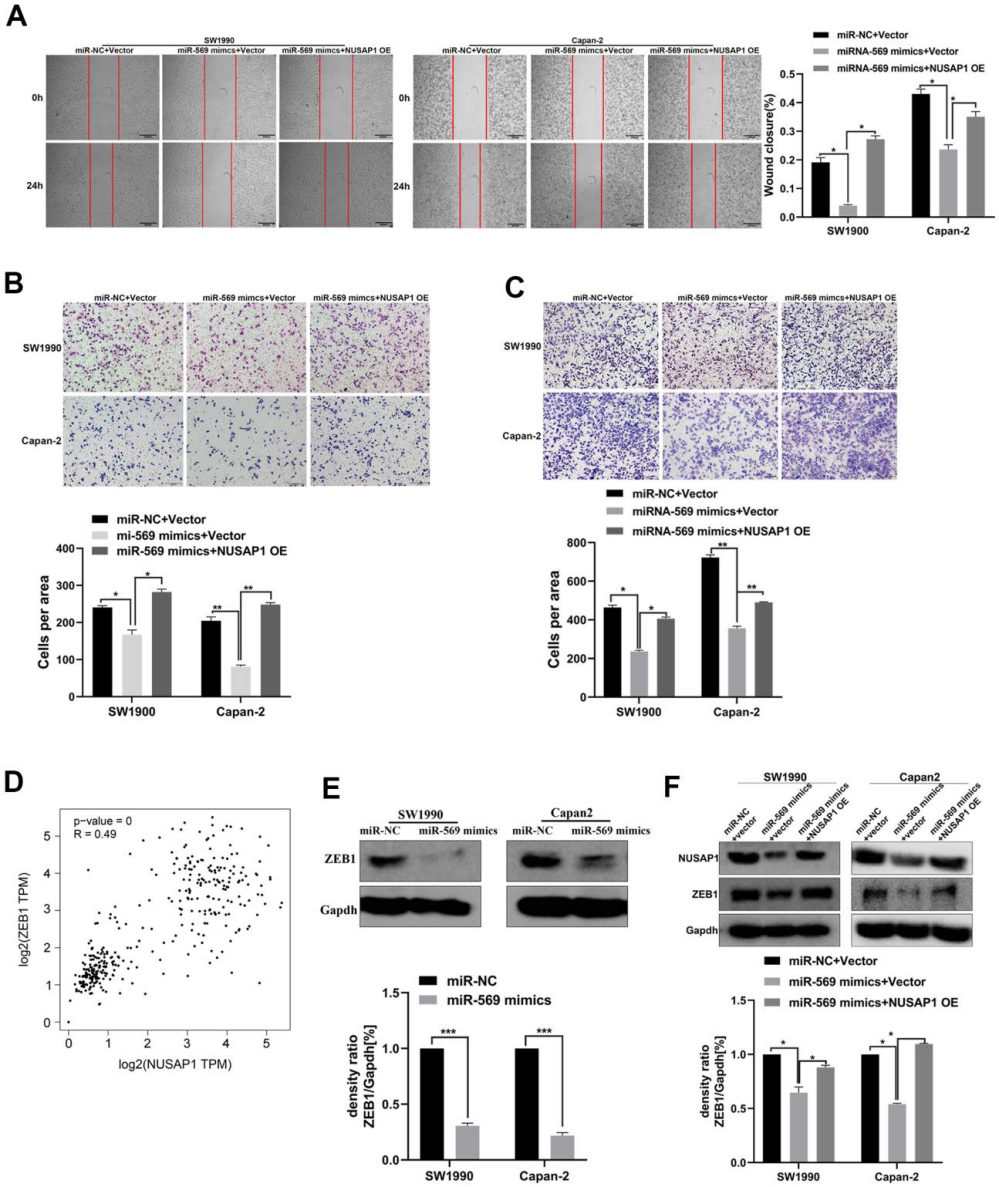
**miR-569/NUSAP1/ZEB1 axis involved in the metastasis of PC cells.** (**A**) Wound healing showed the migratory abilities of PC cells transfected with a combination of miR-NC and vector, or miR-569 mimics and vector, or miR-569 mimics and NUSAP1 OE; (**B**) Transwell assay showed the migratory abilities of PC cells transfected with a combination of miR-NC and vector, or miR-569 mimics and vector or miR-569 mimics and NUSAP1 OE; (**C**) Transwell assay demonstrated the invasive abilities of PC cells transfected with a combination of miR-NC and vector, or miR-569 mimics and vector or miR-569 mimics and NUSAP1 OE; (**D**) The GEPIA database showed that a significant positive correlation between NUSAP1 and ZEB1 could be observed in PC tissues. (**E**) Western blot assay showed the ZEB1 protein expression level after over-expression of miR-569. (**F**) Western blot assay showed the protein expression levels of PC cells transfected with a combination of miR-NC and vector, or miR-569 mimics and vector or miR-569 mimics and NUSAP1 OE; (* *p <* 0.05, ** *p <* 0.01, *n* = 5, Student’s *t*-test, means ± 95% CI).

A functional rescue experiment was performed to further determine whether miRNA-569 inhibits the metastasis of PC cells by targeting NUSAP1. As shown in Figure 8A, 8B, NUSAP1 over-expression reversed the suppression of miRNA-569 up-regulation on PC cell migration. Meanwhile, the reintroduction of NUSAP1 reversed the repression of miRNA-569 on PC cell invasion (Figure 8C). Those results showed that NUSAP1 was involved in mediating the inhibitory effect of miRNA-569 on tumor metastasis. Zinc finger E-box binding homeobox 1 (ZEB1), was also related to tumor metastasis by promoting cell migration and invasion [20]. The GEPIA database showed a significantly positive correlation between NUSAP1 and ZEB1 in PC tissues (Figure 8D). We speculated that miRNA-569/NUSAP1 probably changes the aggressiveness of PC cells by co-regulating ZEB1. As expected, Western blot assay showed that miRNA-569 up-regulation decreased ZEB1 protein levels, whereas over-expressing NUSAP1 restored ZEB1 expression (Figure 8E, 8F). Collectively, these data suggested that NUSAP1 can promote metastasis via ZEB1 up-regulation, while the NUSAP1/ZEB1 axis can be inhibited by miRNA-569.

## DISCUSSION

PC remains one of the deadliest cancer in the world, mainly because most patients are already in the advanced stage when diagnosed [21]. Therefore, exploring new biomarkers and clarifying the potential mechanism of PC progression is very important to developing new PC treatments. An increasing number of literature reports that miRNA can perform a key function in cancer as onco-miRNAs or tumor-inhibited miRNAs during the development of PC [22, 23]. According to a previous study, microRNA-569 expression increased because of 3q26.2 amplification in epithelial cancers, which down-regulated TP53INP1 mRNA expression and enhanced the sensitivity of tumor cells to cisplatin [24]. Zheng et al. revealed that microRNA-569 downregulation occurred in lung cancer, while microRNA-569 overexpression leads to decreased proliferation and migration ability, inducing cell cycle arrest and apoptosis [13]. In addition, microRNA-569 can also be sponged by circ_0001721 to participate in the progression of osteosarcoma [25]. First of all, we observed that miRNA-569 downregulation predicted a poor prognosis in PC. MiRNA-569 was found to act as a suppressive miRNA in PC by directly targeting NUSAP1 to down-regulate ZEB1 expression, thus impeding PC metastasis and progression. These data indicated that microRNA-569 may be a potential therapeutic target for PC.

NUSAP1, a protein binding with microtubules and chromatin, can unite DNA with mitotic spindles and promote microtubule cross-linking during mitosis. It has been reported that NUSAP1 is abnormally expressed in various cancers [26–28]. Li et al. found that NUSAP1 accelerated epithelial-mesenchyme transition (EMT) progression and enhanced cancer stem cell (CSC) signature through the Wnt/β-catenin signal pathway, which led to cervical cancer [26]. Moreover, by activating the TGF-β signaling pathway, NUSAP1 can enhance the proliferation, migration, invasion, and chemotherapy resistance of bladder cancer cells [29]. Furthermore, several previous researches revealed that microRNAs can target and regulate NUSAP1 expression [30, 31]. In our study, we found that NUSAP1 is highly expressed in pancreatic cancer, which is an indicator of poor prognosis. NUSAP1 promoted tumor cell migration and invasion, while microRNA-569 over-expression could reverse this effect by directly binding with its 3’-UTR. These findings suggested that the microRNA-569/NUSAP1 axis functions as a pivotal role in the PC progression.

ZEB1, the member of the ZHF family, could regulate the expression of multiple oncogenes, thereby promoting the initiation and development of cancer [32, 33]. ZEB1 also was the common and important target of a range of signaling pathways (including miRNA signaling) which can regulate cell differentiation, proliferation, plasticity, and survival [34]. For instance, the hepatocyte growth factor activates ERK/MAPK-ZEB1 signal axis to enhance the invasion ability of prostate cancer cells [35]. In the process of bone metastasis of lung cancer, ZEB1, as the downstream target of Wnt/β-catenin, leads to the decrease of E-cadherin expression, which further aggravates EMT [36]. ZEB1 expression was regulated by the well-known transcription factor GRHL2, which can form a double-negative feedback loop with the miR-200 family and ZEB1 [37]. In this study, the microRNA-569/NUSAP1 axis was found to be involved in the process of PC migration and invasion, partly by regulating the ZEB1 signaling pathway. Furthermore, a previous study showed that NUSAP1 contains a DNA binding domain, so it is possible that NUSAP1 acts directly as a transcriptional regulator [38], so we speculated that NUSAP1 protein may directly bind to the ZEB1 promoter and suppress the transcription and promotional activity of ZEB1; however, the binding pattern (direct or indirect) and regulatory mechanism of NUSAP1 and ZEB1 require further experimental verification, perhaps by chromosome co-precipitation or immunoprecipitation.

To sum up, we firstly identified the biological role and regulatory mechanism of miRNA-569 during PC carcinogenesis. Our data concluded: miRNA-569 modulates the NUSAP1/ZEB1 signaling axis, exert anti-tumor function, which is expected to be the clinical therapeutic target of pancreatic cancer.

## MATERIALS AND METHODS

### Cell cultures and tissue samples

The Capan-2(#SUER0449) and SW1990(#TCHu201) were acquired from Suer Biological Technology (Shanghai, China) and the Type Culture Collection of the Chinese Academy of Sciences (Shanghai, China), respectively. Both the cell lines were cultured in DMEM containing 10% FBS, 100 units/ml penicillin-streptomycin at 37° C containing 5% CO2. PC tissue specimens were gathered from the Department of Pathology, the Affiliated Shengjing Hospital, China Medical University, and confirmed by histopathological examination by two pathologists. More detailed patient information has been described in the earlier paper [39].

### RNA isolation and RT-PCR

Total RNA of cultured cells was extracted with Trizol reagent according to the manufacturer’s instructions, and the RNA was stored at -80° C. The concentration and purity of RNA were measured (RNA purity =A260/A280), and the One Step PrimeScript® miRNA cDNA Synthesis kit was used for reverse transcription (RT). Quantitative real-time PCR was performed on ABI 7500 Real-time PCR system (Applied Biosystems) using SYBR Premix Ex Taq II. All the reactions were performed for triplicates. Primer sequences were listed in Table 1.

**Table 1:** Primer sequences.

**Name**	**Forward primer (5'- >3')**	**Reverse primer (5'- >3')**
miR-569	CCCGTAATGAATCCTGGAAAGT	
U6	GCTTCGGCAGCACATATACTAAAAT	CGCTTCACGAATTTGCGTGTCAT
NUSAP1	CAGCCCATCAATAAGGGAGGG	AGTGACCCCTTCAGACCCAA
ZEB1	CAATGATCAGCCTCAATCTGCA	CCATTGGTGGTTGATCCCA
18S	CCCGGGGAGGTAGTGACGAAAAAT	CGCCCGCCCGCTCCCAAGAT

### Transient transfection

NUSAP1 siRNA was designed and synthesized by GENEWIZ (Beijing, China). The NUSAP1 plasmid was purchased from GeneChem (Shanghai, China). miRNA-569 mimics and its control were synthesized and gained from RiboBio (Guangzhou, China). Cells were transiently transfected *in vitro* using jetPRIME reagent (Polyplus) according to protocol.

The siRNA sequences of NC and NUSAP1 were listed:

siNUSAP1-1: 5’-GGAAGACUCUCUGUGGUUTT-3’

siNUSAP1-2: 5’-CCAAGACUCCAGCCAGAAATT-3’

NC siRNA: 5’-AATTCTGCGAACGAGTCACGT-3’

As shown in Supplementary Figure 1, siNUSAP1-1 was more efficient than siNUSAP1-2, which was selected for the follow-up experiments.

### Scratch assay

When the transfected cells reached sufficient confluency, the cell monolayers were scratched across with a 200-ul pipette tip to create a linear wound. Then the supernatant at each well was replaced with a fresh medium without FBS. Migration images were captured at 0, 24 h after scratching by using an inverted bright-field microscope.

### Migration and invasion assay

As for the migration assay: 200 &mu;L serum-free medium containing 3×10^4^ cells was placed in the upper chamber, and 500 &mu;L medium containing 10% fetal bovine serum was added to the lower chamber. As for the invasion assay: the Matrigel was diluted with precooled serum-free medium at a ratio of 1:30, and added 50 &mu;L into the upper chamber. Other procedures were the same as the migration assay. 24 hours later, the chamber was taken out and fixed with 75% ethanol. After staining with Reyes-Giemsa, put it under a fluorescence microscope (Olympus, Tokyo, Japan) for observation and take photos.

### Western blot analysis

Western blot was conducted as previously described [40]. The primary antibodies used are as follows: anti-NUSAP1 (#ab137230), anti-ZEB1(#4650), anti-GAPDH (#25778). Horseradish peroxidase coupled goat anti-rabbit secondary antibody was diluted in TBST at 1:2000. Enhanced chemiluminescence reagent was applied to detect protein bands. Finally, Western blot results were analyzed by NIH Image J software.

### Dual-luciferase reporter assay

Dual-luciferase reporter assays were performed as we previously described [39]. Briefly, NUSAP1 3’UTR containing the predicted wild-type (WT) or mutated (Mut) binding sites of miR-569 were cloned into the pGL3 vector. At 48h after co-transfection of miR-569 mimics and luciferase reporter vector into cells, luciferase activity was detected.

### *In vivo* metastasis assay

Female BABL/c nude mice (n=10, 4–6 weeks old) were got from Vitalriver (Beijing, China). Capan-2 cells (1×10^6^) were labeled with luciferase ahead of time. After transfection with agomir-NC (5μM) or agomir-569 (5μM), transfected cells were injected into the mouse spleen (n=5 in each group), respectively. 10 weeks after injection, the mice were killed according to the requirements, and the liver tissues were collected and embedded in paraffin. Then HE staining and pathological analysis was performed. The relevant experimental scheme and content were approved by the Ethics Committee of China Medical University (Approval no. 2020322).

### Bioinformatics

The public database TargetScan (http://www.targetscan.org/vert_72/) was used to determine the potential miR-mRNA interactions. The predicted target genes are intersected with aberrantly expressed data from the TCGA portal (http://tumorsurvival.org/) database displayed by a Venn diagram. GO annotation and KEGG pathway of target genes were performed by DAVID 6.8 software (https://david.ncifcrf.gov/). The results were visualized with the “clusterProfiler _3.11.0” package of the R 3.6.0 language. PPI networks were generated by STRING 11.0 (http://string-db.org), which aimed to assess and integrate proteins from prediction or experiments. The interaction network was visualized by Cytoscape 3.8.1 and the MCODE plugin was conducted to screen potential clusters. In the process of selecting model parameters, the minimum absolute contraction and LASSO regression methods are widely used. The “glmnet 4.1.2” package was selected for modeling research. The Oncomine database (http://www.oncomine.org) was applied to analyze the transcription expression level of the NUSAP1.

### Statistical analysis

GraphPad Prism 8.0.1 and R 3.6.0 were selected to analyze the experimental data, and presented as the means ± standard deviations (SD). All experiments were carried out in triplicate. Group comparison was performed by Student’s t-test and the threshold of significant difference was p<0.05.

## Supplementary Material

Supplementary Figure 1
