# Supplementary figures and images for: microRNA-569 inhibits tumor metastasis in pancreatic cancer by directly targeting NUSAP1
Source: Aging (Albany NY). 2022 Apr 28;14(8):3652–65. doi: 10.18632/aging.204035 (PMC9085231; doi:10.18632/aging.204035)

## SUPPLEMENTARY FIGURE

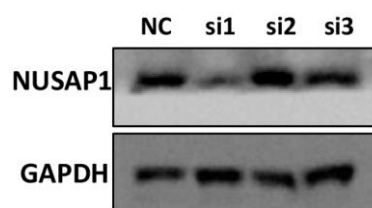

Supplementary Figure 1. Western blot of NUSAP1 knockdown.

Supplement: Supplementary Figure 1 [file aging-14-204035-s001.pdf]
